# Lipid Droplet‐Derived Biomimetic Nanocarriers for the Enhancement of Porcine Intermuscular Fat Content

**DOI:** 10.1002/advs.202406150

**Published:** 2025-05-14

**Authors:** Pengxiang Zhao, Hongbo Han, Jingjie Hao, Ziwei Yu, Zichen Zhao, Lupeng Chen, Heng Wang, Jian Wu, Zhuqing Ren

**Affiliations:** ^1^ Key Laboratory of Agriculture Animal Genetics Breeding and Reproduction of the Ministry of Education College of Animal Science Huazhong Agricultural University Wuhan Hubei 430070 P. R. China; ^2^ College of Animal Science and Technology Shandong Agricultural University Taian 271017 P. R. China; ^3^ Frontiers Science Center for Animal Breeding and Sustainable Production Wuhan 430070 P. R. China; ^4^ Hubei Hongshan Laboratory Wuhan 430070 P. R. China

**Keywords:** intermuscular fat, lipid droplets, nanocarriers, pork

## Abstract

Intramuscular fat (IMF) content is a critical factor influencing the quality and palatability of pork. However, established breeding and nutritional methods lead to systemic fat deposition. The efficient and specific methods to increase IMF content in livestock remain a challenge. In this study, Lipid droplets (LDs), an organelle in cells, are shown to be a controllable and biocompatible carrier that can be used to increase IMF content. This study demonstrated that isolated LDs stimulate lipid synthesis by encapsulating Oleic Acid (OA), a PPARγ ligand. Importantly, artificial LDs (aLDs) can effectively substitute isolated LDs. In vivo, investigations showed that OA‐loading aLDs significantly increased the expression of lipid synthases such as DGAT2 and FASN through activation of PPARγ, a key pathway for lipid synthesis. Furthermore, the IMF content on the injected side is increased up to 35% with minimal side effects. The development of OA‐aLDs promotes IMF deposition and demonstrates the significant potential of aLDs as drug carriers. The exploration of LDs‐based carriers provides a new theoretical basis for their application in the medical field.

## Graphical abstract

The article presents the development of artificial lipid droplets (aLDs) as biomimetic nanocarriers to enhance intramuscular fat (IMF) content in pigs. These aLDs, loaded with oleic acid, significantly increase IMF by up to 35% in muscle tissues without adverse side effects. This innovative approach improves pork quality by enhancing marbling, offering economic benefits for the meat industry.

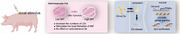

## 1 Introduction

In livestock husbandry, adipocytes are abundantly distributed within subcutaneous and mesenteric tissues, with deposits also observed in the peripheral tissues of the viscera, and a portion dispersed between or within the muscle fibers.^[^ ^1^ ^]^ While subcutaneous and visceral adipose deposits typically have diminished commercial value, the distribution extent of Intramuscular Fat (IMF) deposition has a significant influence over the flavor and palatability of meat. Elevated IMF deposition results in the formation of white streaks, commonly recognized as “marbling”.^[^ ^2^ ^]^ IMF content significantly impacts the sensory attributes of pork, such as tenderness, juiciness, and flavor. These qualities are paramount in consumer satisfaction and market value.^[^ ^3^ ^]^ Consumers are more likely to purchase pork products with high IMF content.^[^ ^4^ ^]^ Therefore, increasing IMF content while reducing subcutaneous and visceral adiposity, without compromising carcass leanness, is a common goal for both porcine genetic breeders and feed managers.^[^ ^5^ ^]^ This overarching goal is pursued through two principal avenues: genetic improvement and nutritional intervention.^[^ ^6^ ^]^ However, current methods lead to overall adiposity deposition in livestock, and increasing backfat thickness, and do not specifically target an increase in IMF contents.

The formation of IMF is a tightly regulated process involving genetic, molecular, and environmental interactions. The differentiation of preadipocytes into mature adipocytes plays a key role. This process is regulated by transcription factors such as PPARγ and C/EBPs, which activate genes that drive lipid accumulation and storage.^[^ ^7^ ^]^ The essence of IMF deposition involves the accumulation of Lipid Droplets (LDs) within adipocytes or embedded within muscle fibers. These adipocytes provide the site for marbling genesis.^[^ ^8^ ^]^ Intermuscular adipocytes are mainly found between primary and secondary muscle bundles encircling bovine and porcine anatomies. Marbled adipocytes are also present within muscle bundles of top‐tier grades in Japanese Black Cattle.^[^ ^9^ ^]^ The abundance and dimensions of LDs within adipocytes have a significant impact on IMF content. LDs, constituting ubiquitous organelles within adipocytes, play a pivotal role in lipid metabolism.^[^ ^10^ ^]^ LDs serve as repositories for intracellular lipid storage and trafficking.^[^ ^11^ ^]^ Composed of a lipid core and phospholipid monolayer, they provide a lipid buffering mechanism in response to metabolic fluctuations and interact with other subcellular compartments to maintain physiological homeostasis.^[^ ^12^ ^]^ Multiple transcription factors are involved in regulating the biosynthesis and metabolism of IMF.^[^ ^13^ ^]^ Particularly, PPARγ plays a central role in the regulation of LDs and IMF formation.^[^ ^14^ ^]^ It promotes adipocyte differentiation and activates the expression of lipid synthesis‐related genes, such as FABPs, plin1, and DGATs. In multiple porcine species, the expression level of PPARγ was positively correlated with IMF content, with an increase in IMF accompanying enhanced PPARγ expression. Muscle‐specific overexpression of PPARγ pigs has been constructed using CRISPR/Cas9. PPARγ‐overexpressing pigs showed a significant increase in IMF content without a change in lean body mass. This study provided new theoretical support for breeding high IMF pigs.^[^ ^15^ ^]^

Nanolipid particles are an important tool in the field of drug delivery. They can encapsulate drugs, increase their bioavailability, extend plasma half‐life, and reduce toxic side effects.^[^ ^16^ ^]^ Researchers improve the preparation of nanolipid particles to enhance their biological safety and stability.^[^ ^17^ ^]^ Interestingly, the structure of nanolipid particles is similar to that of LDs, both containing a lipid matrix and surfactant.^[^ ^18^ ^]^

Inspired by these attributes, we hypothesized that LDs could be designed as a conceivable delivery vehicle to enhance IMF content by directly increasing LD abundance in muscle tissue via injection.^[^ ^19^ ^]^ Additionally, the lipophilic nature of LDs provides an ideal reservoir for metabolic activators, stimulating lipid synthesis pathways to enhance the efficacy of intervention. In addition, LDs are easily separated from other cellular components and are available in large quantities, which may pave the way for large‐scale production.^[^ ^19^ ^]^ Here, we demonstrate that isolated LDs loaded with Oleic Acid (OA), can effectively enhance IMF content. Notably, we can fabricate stable and manageable artificial LDs (aLDs) through in vitro synthesis. These aLDs demonstrate pronounced effectiveness and biological potency within a physiological environment, with minimal impact on normal organ function. This breakthrough has significant implications for livestock production and exhibits prospective utility in clinical translation.

## 2 Results

### 2.1 Isolation and Characterization of LDs

**Figure 1:**
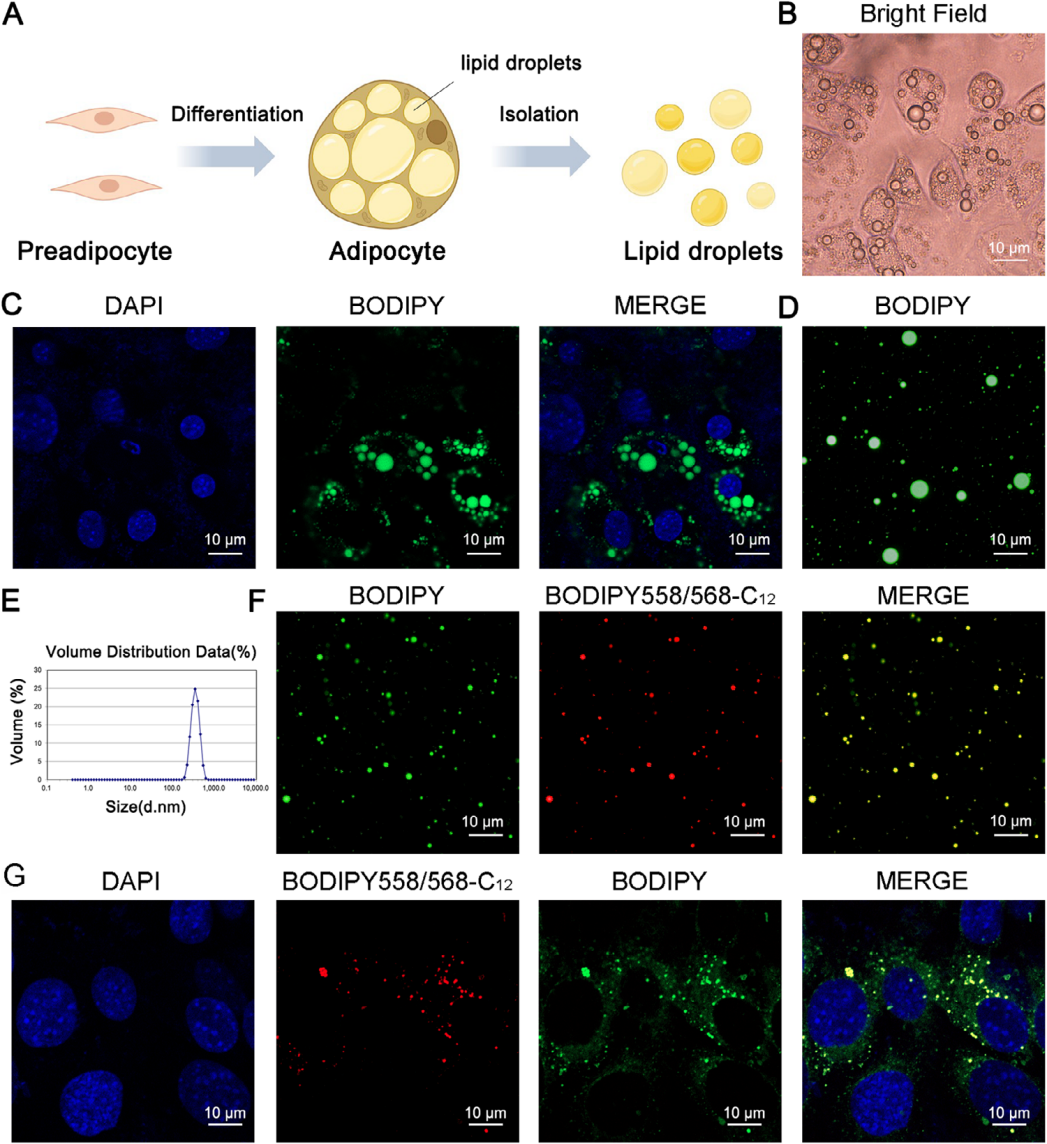
Isolation and characterization of lipid droplets. A) Schematic diagram of preadipocyte differentiation and extraction of lipid droplets (LD = lipid droplet). B) Bright‐field observation of differentiated cells. Scale bar: 10 µm. C) Fluorescent staining of induced differentiated cells. Scale bar: 10 µm. D) Fluorescent staining to examine the morphology of isolated LDs. Scale bar: 10 µm. E) Particle size of isolated LDs determined by DLS measurement. F) Isolated LDs are able to act as a reservoir for taking up fatty acids, which LDs were stained with BODIPY 558/568‐C_12_ (Red). Scale bar: 10 µm. G) Fluorescent staining of isolated LDs followed by incubation of cells. Scale bar: 10 µm.

To obtain adipocytes containing a large number of LDs, we first induced C3H10 preadipocytes to differentiate into adipocytes in vitro (**Figure** **1**A), during which spherical LDs gradually matured (Figure 1B). BODIPY 493/503 is a neutral lipid‐specific dye that is widely used to stain LDs in cells. It had a high affinity for hydrophobic, lipid‐rich environments while avoiding significant background staining. This property makes it an excellent tool for imaging and quantifying LDs.^[^ ^20^ ^]^ The formation of LDs within adipocytes was further validated through confocal imaging after staining LDs with BODIPY (Figure 1C). LDs were isolated from adipocytes by a previously reported method and their structural integrity was assessed using BODIPY staining.^[^ ^21^ ^]^ Confocal images showed that the isolated LDs maintained favorable morphology (Figure 1D). Dynamic light scattering measurements showed the LDs' diameter was determined to be 355±27.3 (Mean ± SD) nm (Figure 1E). Subsequently, we demonstrated that isolated LDs could persist stably in Phosphate Buffer Solution (PBS) for 7 days without significant changes in size and morphology. This stability provided the possibility for their further utilization (Figure S1, Supporting Information).

OA is a commonly found unsaturated fatty acid and has been reported to be ligands for PPARγ, whose activation precipitates lipid synthesis within organisms.^[^ ^22^ ^]^ We hypothesized that LDs could be engineered as a delivery system to specifically increase IMF content by activating PPARγ in vivo and promoting lipid synthesis through loading OA. For this, we investigated the localization of exogenously added fatty acids in endogenous LDs. We co‐cultured the BODIPY558/568‐labeled fatty acid C_12_ with isolated LDs for 30 min, and confocal microscopy imaging showed efficient localization of C_12_ with LDs (Figure 1F). Similarly, after co‐incubation with the C3H10 cell for 16 h, we detected the C_12_ fluorescent probe in the intracellular LDs (Figure 1G). Thus, we demonstrated that fatty acids could be specifically loaded into LDs after adipocyte differentiation in vitro. For a better description, we named “OA‐loaded LDs (OA‐LDs)” as LDs containing OA after incubation.

### 2.2 Uptake and Distribution of LDs in C2C12 Cells

**Figure 2:**
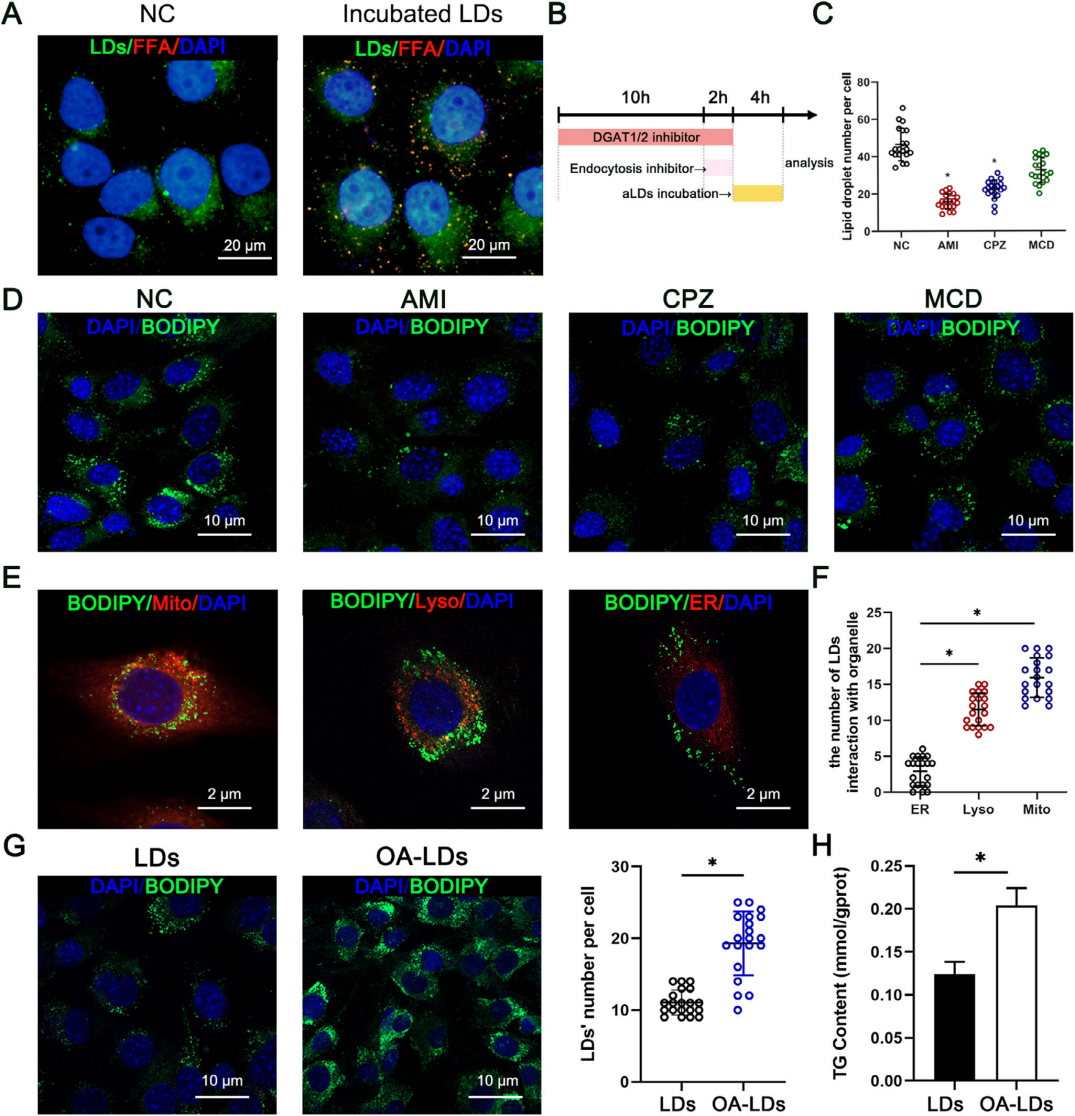
Uptake and distribution of LDs in C2C12 cells. A) Fluorescent staining of fatty acid‐labeled LDs incubated with cells. Scale bar: 10 µm. B) Schematic diagram of the process of incubating different endocytosis inhibitors. C) Counting of cellular LDs after incubation with different inhibitors. ^*^ *p* < 0.05. D) Confocal images of LDs after incubation with different inhibitors. Scale bar: 10 µm. E) Confocal images of LDs distribution after incubation with other organelle. Mitochondria, Lysosome, and ER were stained with Mitotracker Red, LysoTracker Red, and ER‐Tracker Red, respectively. Scale bar: 10 µm. F) Counting of LDs interacted with other organelle. *n* = 20, ^*^ *p* < 0.05. G) Confocal images and LDs counting of cells after incubation with LDs or OA‐LDs. *n* = 20, ^*^ *p* < 0.05. H) Triglyceride content of cells after incubation with LDs or OA‐LDs. *n* = 3, ^*^ *p* < 0.05.

Before verifying the enhancement effect of OA‐loaded LDs on IMF content, we investigated the uptake mechanism and distribution of LDs in C2C12 cells. BODIPY 493/503 was used to label the total LDs in the cell. BODIPY558/568‐labeled fatty acid C_12_‐labeled LDs were incubated with cells and intracellular localization could be observed after 4 h, indicating the entry of LDs into the cells (**Figure** **2**A). We then investigated the pathway of LDs uptake into the cells. The strategy involved the inhibition of cellular lipid synthesis utilizing DGAT1/2 inhibitors (A922500 and PF‐06424439), effectively curtailing the intracellular content of LDs before incubating LDs. C2C12 cells were then treated with different inhibitors of endocytosis to investigate the mechanism of lipid droplet uptake (Figure 2B). Notably, confocal imaging showed a noteworthy reduction in uptake efficiency upon pre‐treatment of cells with clathrin‐mediated endocytosis inhibitor (chlorpromazine, CPZ) and microprotein phagocytosis inhibitor (amiloride, AMI). Conversely, no significant alterations in cellular uptake were observed in cells pre‐treated with lipid raft inhibitor (methyl‐β‐cyclodextrin, MCD) (Figure 2C,D).

To investigate the distribution of LDs in cells, different intracellular compartments, including lysosomes, mitochondria, and endoplasmic reticulum, were stained with the corresponding fluorescent probes, followed by cluster analysis using confocal microscopy. Fluorescence co‐localization reflects the contact of LDs with organelles. It was revealed that LDs had more contact with lysosomes and mitochondria, while less contact with the endoplasmic reticulum (Figure 2E,F). This indicated that the isolated LDs were bioactive and able to interact with organelles after re‐incubation. We next evaluated the effect on enhancing lipid content. After 4 h of co‐incubation with OA‐loaded LDs, confocal imaging analysis showed a significant increase in the number of intracellular LDs (Figure 2G). The cellular triglyceride content was also significantly increased after incubation with OA‐LDs (Figure 2H). These results suggested that LDs were able to enter cells through clathrin‐mediated endocytosis or microprotein phagocytosis and maintain multiple interactions with intracellular organelles. More importantly, LDs loaded with OA significantly increased cellular lipid content.

### 2.3 OA‐Loaded aLDs Preparation and Concentration Selection

**Figure 3:**
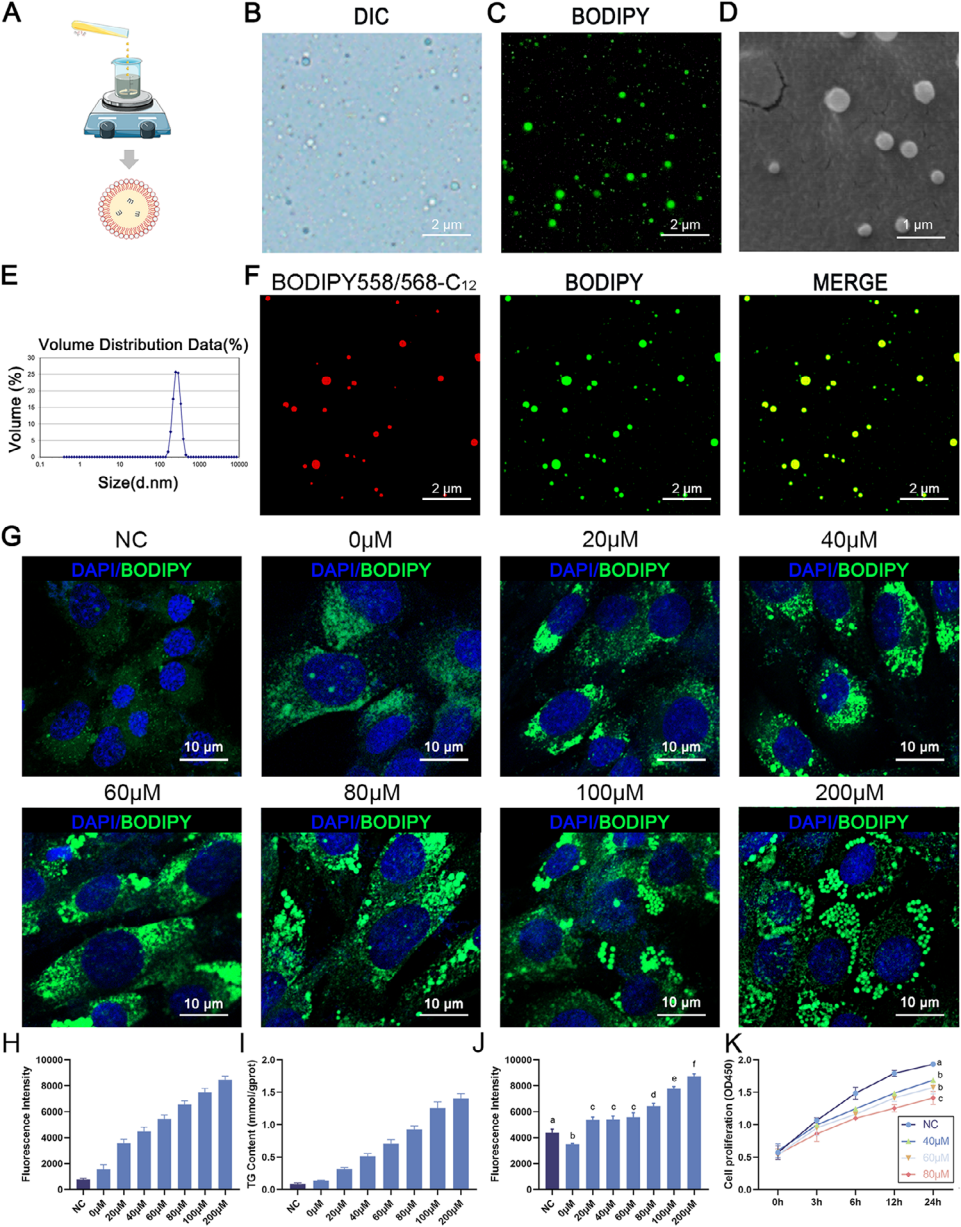
OA‐Loaded artificial lipid droplet preparation and concentration selection. A) Schematic diagram of the preparation of artificial lipid droplets (aLDs). B) Phase contrast microscopy of aLDs. Scale bar: 2µm. C) Fluorescence staining of aLDs by BODIPY493/503. Scale bar: 2µm. D) Scanning electron microscopy of aLDs. Scale bar: 1 µm; E) Particle size of aLDs determined by DLS measurement. F) aLDs could take up fatty acids, which LDs were stained with BODIPY 558/568‐C_12_ (Red). Scale bar: 2 µm. G) Confocal images of C2C12 after incubation with concentration gradients of OA‐aLDs. Scale bar: 10 µm. H) Fluorescence intensity of C2C12 cell after incubation with concentration gradients of OA‐aLDs. *n* = 3. I) TG content of C2C12 cell after incubation with OA‐aLDs. *n* = 3. J) The ROS detected C2C12 cells after incubation with OA‐aLDs. *n* = 3, p<0.05. K) Cell activity of C2C12 cell after incubation with OA‐aLDs. *n* = 3, ^a, b, c^, *p* < 0.05.

The preceding findings demonstrate the competence of LDs as a delivery vehicle for fatty acids, which led to an increase in cellular lipid content. However, the time and economic cost accompanying LD isolation from cells proved untenable for livestock production. Hence, an expedient and efficient alternative became imperative. We previously reported a method for the preparation of artificial LDs (named “aLDs”)^[^ ^23^ ^]^ and we prepared aLDs for subsequent investigations. Briefly, the aLDs were prepared by dissolving DOPC (1,2‐Di(9z‐octadecenoyl)‐sn‐glycero‐3‐phosphocholine) and TAG with ethanol, followed by mixing with PBS under sufficient stirring (**Figure** **3**A). The prepared aLDs were observed as white spheres under phase contrast microscopy (Figure 3B), and neutral lipid‐specific staining showed that the aLDs maintained the same structure as the endogenous LDs (Figure 3C). Morphological scrutiny through scanning electron microscopy unveiled spherical aLDs with a non‐adherent surface (Figure 3D). Their particle size distribution was determined to be 322 ± 36.2 nm (Figure 3E). Significant co‐localization could be observed with the addition of BODIPY558/568‐labeled C_12_ during preparation (Figure 3F), indicating that the aLDs had the same fatty acid storage function as endogenous LDs.

We next introduced a concentration gradient of OA during aLDs preparation, yielding OA‐loaded aLDs (named OA‐aLDs) across diverse concentrations. The encapsulation and release efficiency were 93.8 ± 1.3% and 11.66 ± 0.8%, respectively. In vitro release efficiency assays revealed a rapid release rate of OA in the first 24 h, followed by stabilization (Figure S2, Supporting Information). Subsequently, intracellular lipid content was measured after incubating OA‐aLDs for 4h. Confocal image analysis showed a positive correlation between the fluorescence intensity of LDs and OA concentration (Figure 3G,H). As the OA concentration increased, the same trend was observed for the cellular triglyceride content, showing an incremental increase (Figure 3I).

Excessive intake of fatty acids can induce lipotoxicity and cellular stress. In this study, we aimed to elevate IMF content rather than cause stress and inflammation. Therefore, an assessment of cellular stress induced by OA‐aLDs was imperative. Reactive oxygen species (ROS) levels were measured after OA‐aLDs were treated with DCFH‐DA. The results showed that more intense cellular stress was induced when the concentration of OA exceeds 80 µm (Figure 3J). Notably, the cells died under strong stress when the OA concentration exceeded 200 µm. Cellular stress could also cause a decrease in proliferative activity. Thus, the effect of OA‐aLDs on cell proliferation was explored. Treatments with OA concentrations above 80 µm and below 200 µm were excluded because of excessively strong ROS and low lipid synthesis capacity. Similarly, cell proliferation capacity decreased with increasing OA concentration (Figure 3K). Consequently, we opted for 60 µm OA‐aLDs for subsequent experiments within this study, given its strong lipid synthesis stimulation and the relatively lower imposition of cellular stress.

### 2.4 aLDs Promote OA Uptake and Stimulate Lipid Synthesis

**Figure 4:**
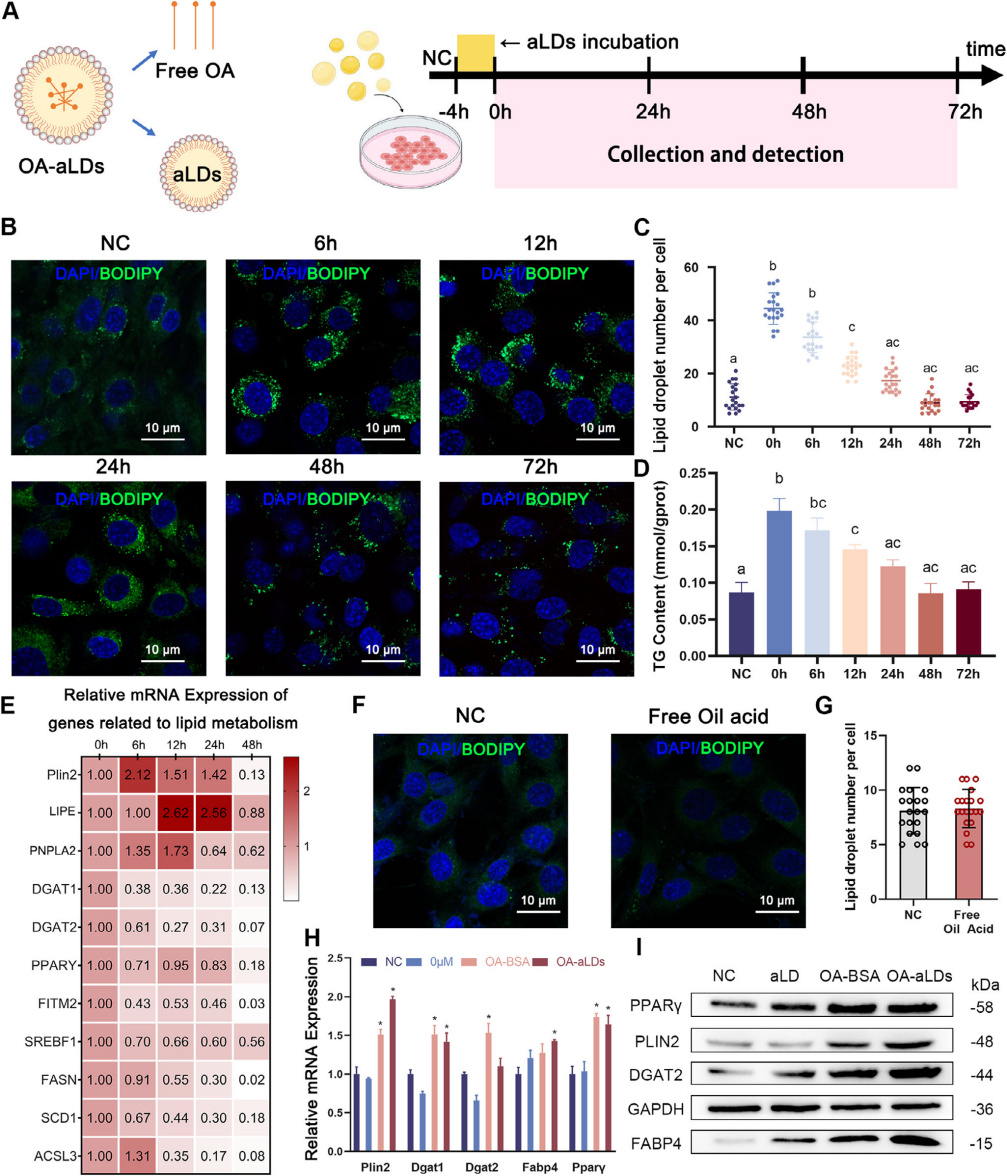
aLDs promote OA uptake and stimulate lipid synthesis. A) Schematic diagram of the uptake of aLDs. B, C) Confocal images and counting of LDs number after incubation of aLDs in C2C12 cell at different times. *N* = 20, Scale bar: 10 µm. *p* < 0.05. D) TG content of C2C12 cell after incubating with aLDs. *n* = 3, *p* < 0.05. E) Relative mRNA expression of genes related to lipid metabolism in C2C12 after incubated aLDs. F, G) Confocal images and counting of LDs number after incubating free OA in C2C12 cell for 4h. Scale bar: 10 µm. n = 20. H) Relative mRNA expression of genes related to lipid metabolism in C2C12 after different treatments. *n* = 3, ^*^ *p* < 0.05. I) Detection of genes and pathways related to lipid synthesis after incubation with OA‐aLDs by Western Blot.

We measured the effects of aLDs on intracellular lipid content. The number of LDs in the cells was counted at different time intervals after incubating the cells with aLDs (**Figure** **4**A). Fluorescent staining revealed a peak LD count at 0 h, followed by a progressive decrease over time. This reduction indicated aLDs consumption via cellular catabolism subsequent to uptake (Figure 4B). Specifically, a significant reduction in LDs number after 24 h (Figure 4C). Concurrently, the cellular triglyceride content (Figure 4D) and fluorescence intensity (Figure S3, Supporting Information) showed a similar decreasing trend, indicating a transient amplification facilitated by aLDs.

We further investigated the effect of incubating aLDs on genes related to lipid metabolism. The qPCR results showed that the expression of perilipins 2 increased significantly, which was involved in the localization and recognition of aLDs (Figure 4E). Consistent with the decrease in the number of LDs, the expression of Hormone‐Sensitive Lipase (Lipe) and Adipose Triglyceride Lipase (Pnpla2), which are critical genes for lipolysis, was significantly up‐regulated. Similarly, the expression of neutral lipid synthesis genes: Diacylglycerol O‐Acyltransferase 1 or 2(Dgat1 or Dgat2), PPARγ, Fat Storage Inducing Transmembrane Protein 2 (Fitm2) and fatty acid‐related genes: Sterol Regulatory Element Binding Transcription Factor 1 (Srebf1), Fatty Acid Synthase (Fasn), Stearoyl‐CoA Desaturase1 (Scd1), Acyl‐CoA Synthetase Long‐Chain Family Member 3 (Acsl3) were suppressed to varying degrees. These changes in gene expression suggest that lipolysis is activated and lipid synthesis is inhibited after ingestion of aLDs.

A commonly used fatty acid medium employs oleic acid‐modified bovine serum albumin (OA‐BSA) as a stimulant, unlike the free oleic acid (Free‐OA) used in our study. We further evaluated the effect of Free‐OA. The results showed that Free‐OA floated on the surface of the medium due to its strong hydrophobicity and could neither be absorbed by cells nor increase intracellular LDs (Figure 4F,G); therefore, it could not stimulate lipid synthesis. Given the limited efficacy of both aLDs carriers and Free‐OA in increasing cellular lipid content, our attention shifted to evaluating the effect of OA‐aLDs. After incubating OA‐aLDs for 24 h, five lipid synthesis‐related genes that were detected were significantly up‐regulated for expression (Figure 4H), and Western blot demonstrated the same effect (Figure 4I). These results collectively suggested the profound efficacy of aLDs as a delivery carrier, which facilitates the uptake of OA into the intracellular space to stimulate.

### 2.5 OA‐aLDs Enhance Lipid Content in Muscle Tissue

**Figure 5:**
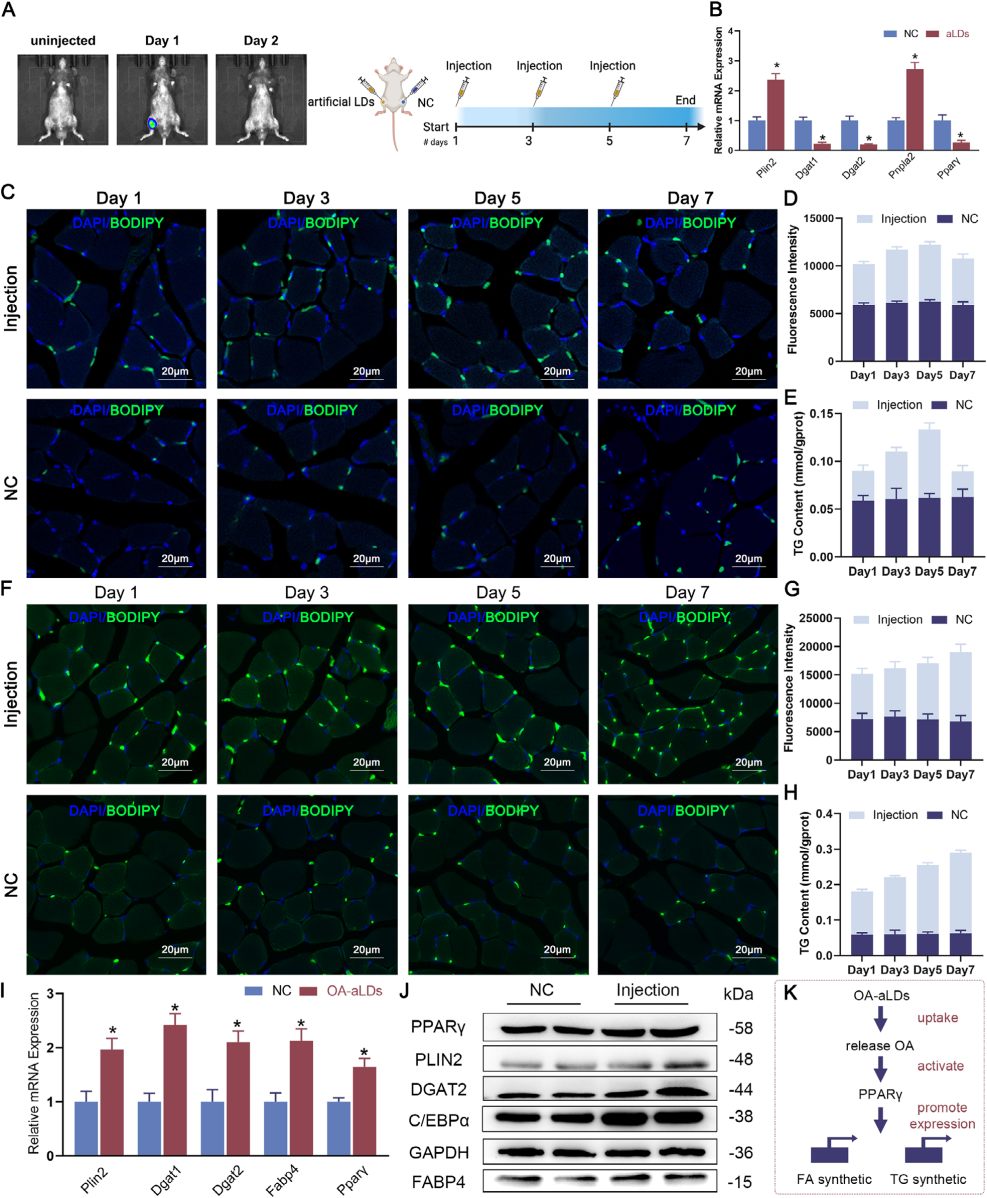
OA‐aLDs enhance lipid content in muscle tissue. A) Live imaging of mice after injection of Rhod‐labeled aLDs and schematic diagram of two‐day injection schedule. B) Relative mRNA expression of genes related to lipid metabolism in gastrocnemius muscle after injected aLDs. *n* = 3, ^*^ *p* < 0.05. C, D) Confocal images and counting of fluorescence intensity after injecting aLDs in gastrocnemius muscle. *n* = 3, Scale bar: 40 µm. E) TG content of gastrocnemius muscle after injection. F, G) Confocal images and counting of fluorescence intensity after injected OA‐aLDs in gastrocnemius muscle. *n* = 3, Scale bar: 40 µm. H) TG content of gastrocnemius muscle after injected OA‐aLDs. *n* = 3. I) Relative mRNA expression of genes related to lipid metabolism in gastrocnemius muscle after injected OA‐aLDs. *n* = 3, ^*^ *p* < 0.05. J) Detection of genes and pathways related to lipid synthesis after injected with OA‐aLDs in gastrocnemius muscle by Western Blot. K) Schematic diagram of the promotion of lipid synthesis by OA‐aLDs.

In vitro assays have unequivocally demonstrated that Free‐OA must be encapsulated within aLDs to effectively serve as a delivery carrier, enabling cellular uptake and enhancing lipid synthesis. We further validated it in vivo with 8‐week‐old male C57BL/6 mice. We injected aLDs into the left gastrocnemius muscle, while physiological saline was injected at the corresponding site on the right side as a control. After a single injection of Rhod‐labeled aLDs, we confirmed the signal persistence of aLDs in tissues by live imaging (**Figure** **5**A Complete results are shown in Figure S4, Supporting Information). The disappearance of rhodamine signal between Day1‐Day2 indicated that aLDs were able to be maintained in the tissues for 24–48 h. Therefore, we set up a two‐day injection routine. Tissue samples were subsequently collected at different time intervals for comprehensive analysis (Figure 5A). Consistent with the results of the in vitro assay, although the expression and localization of the Plin2 gene were increased after injection, the down‐regulated expression of Dgat1, Dgat2, and Pparγ implied that lipid synthesis was blocked in gastrocnemius muscle tissue. Meanwhile, significant upregulation of the Pnpla2 gene indicated enhanced lipid catabolic processes (Figure 5B). We stained LDs in tissues using the specific dye BODIPY493/503. The number of green fluorescent spots was notably increased and extensive after injection (Figure 5C), with the increase of BODIPY fluorescence intensity during the first 5 days (Figure 5D), reflecting an elevated average lipid content of each muscle fiber. Although the TG content of the injected group (0.089 ± 0.005 mmol/gprot) was higher than that of NC (0.062 ± 0.005 mmol/gprot) on day 7, it did not exceed the total amount injected (0.102 ± 0.007 mmol/gprot) (Figure 5E). It's worth noting that Free‐OA also proved ineffective in stimulating lipid synthesis in gastrocnemius muscle tissue, as no significant increase in triglycerides could be detected after injection. This collective evidence suggests that neither aLDs nor Free‐OA can effectively induce lipid synthesis in muscle tissue.

We further investigated the effects of OA‐aLDs using the same strategy. Tissue fluorescence staining revealed more numerous and larger green fluorescent spots after injection. Some LDs had fused into linear formations on Day 7 (Figure 5F). The ratio of BODIPY fluorescence intensity also increased over time, thus indicating an elevated lipid content within the muscle fibers (Figure 5G). The triglyceride content in gastrocnemius muscle tissue consistently increased from Day 1 to Day 7 (Figure 5H). The TG content of the injected group (0.289 ± 0.007 mmol/gprot) increased compared to the control group (0.628 ± 0.008 mmol/gprot) on day 7, exceeding the total lipid content of the OA‐aLDs after 3 injections (0.101 ± 0.009 mmol/gprot). Lipid synthesis was also enhanced, accompanied by a significant upregulation of Plin2, Dgat1, Dgat2, Fabp4, Pparγ, and C/ebpα expression at both mRNA and protein levels (Figure 5I,J). To exclude the harmful effects of OA‐aLDs on the organism, we carried out various detections, including multi‐tissue HE staining, blood cell analysis, and inflammatory factor expression assay. After injection, HE staining of other tissues (Figure S5A, Supporting Information) and blood routine examination (Table S1, Supporting Information) showed no significant abnormalities. The ELISA results did not show a significant increase in the expression of the major inflammatory factors and even a significant decrease in the expression of TNF‐α and IL‐10 (Figure S6, Supporting Information). Similarly, we performed injection experiments using Rhod‐labeled OA‐aLDs. Fluorescent signals were not detected in tissues beyond muscle tissue (Figure S5B, Supporting Information). This suggested that OA‐aLDs did not produce harmful effects and inflammatory responses in multiple organs and tissues. These findings emphasize the potent efficacy of OA‐aLDs as a delivery system to dramatically increase lipid synthesis in vivo. OA enhances PPARγ activity and promotes transcription of downstream genes (Figure 5K).

### 2.6 OA‐aLDs Increased IMF in Longissimus Dorsi of Porcine

**Figure 6:**
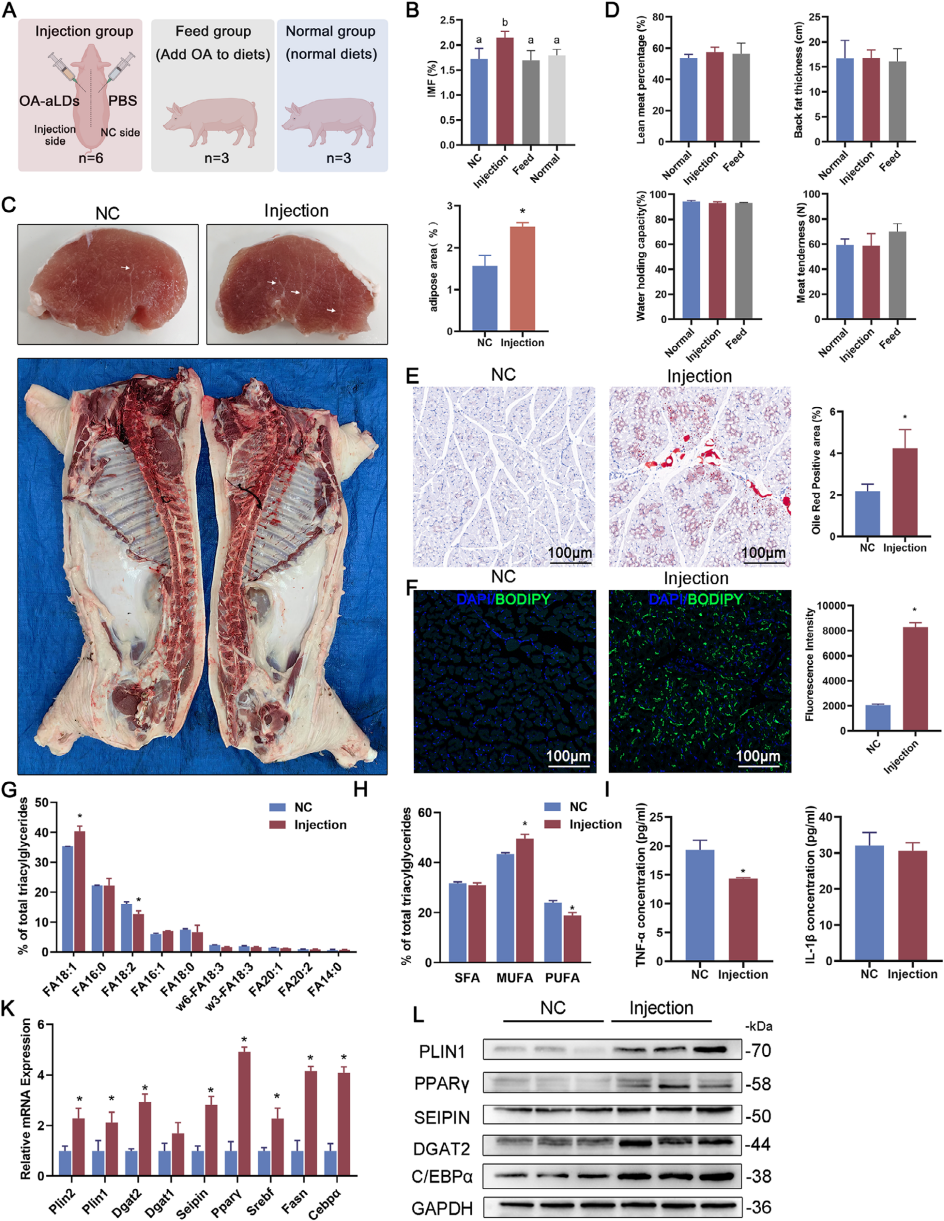
OA‐aLDs increased IMF in longissimus dorsi of Porcine. A. Schematic diagram of the pig injection test. Twelve 100‐day‐old healthy male Tongcheng pigs were used for the experiment. B) Measurement of IMF content in the longest dorsal muscle. ^*^ *p* < 0.05. C) Above: Image of the cross‐section of the longest dorsal muscle after injection. Arrows indicate intramuscular fat deposits. Relative area of adipose was counted by ImageJ, *n* = 3, ^*^ *p* < 0.05. Below: Pork separated longitudinally into two parts along the midline of the spine. Left side, NC; right side, injection. D) Measurement of carcass and meat quality traits. ^*^ *p* < 0.05. E) Oil red O staining of the longest dorsal muscle. Scale bar: 100 µm. *n* = 3, ^*^ *p* < 0.05. F) Confocal images of tissue fluorescence staining in longissimus dorsi. Scale bar: 100 µm. *n* = 3, ^*^ *p* < 0.05. G) Statistical plot of the relative percentage of multiple fatty acid components. ^*^ *p* < 0.05. H) Statistical chart of the proportion of saturated, monounsaturated, and polyunsaturated fatty acids. ^*^ *p* < 0.05. I) Elisa assay of Porcine TNF‐α and IL‐1β content. ^*^ *p* < 0.05. K) Relative mRNA expression of genes related to lipid metabolism in longissimus dorsi after injected OA‐aLDs. ^*^ *p* < 0.05. L) Detection of genes and pathways related to lipid synthesis after injected with OA‐aLDs in gastrocnemius muscle by Western Blot.

Previous studies have demonstrated the high efficacy of OA‐aLDs in enhancing lipid synthesis. We validated the stimulatory effect of OA‐aLDs on IMF in Chinese Tongcheng pigs at 100 days of age because this stage had weaker fat deposition and was better to reflect the changes in IMF content. Injections were performed on Days 1 and 3, and samples were collected on Day 5 (**Figure** **6**A). To investigate the effect of different feeding modes on IMF formation, the Feed group was supplemented with the same mass of OA‐aLDs from the time of injection with a normal diet. Compared to the NC (negative control, normal diet), more fat deposition was observed in muscle after injection, with no significant change in carcass characteristics or meat quality (Figure 6C and Table S3, Supporting Information). The IMF content was significantly increased to 2.1%, a 35% enhancement over the control (Figure 6B). The injections did not induce significant changes in lean meat percentage, backfat thickness, water hold capacity, and meat tenderness (Figure 6D). Oil‐red O staining revealed a significant increase in positive staining areas after injection (Figure 6F). Fluorescence staining also showed more accumulation of LDs after injection, indicating enhanced lipid content in the longest dorsal muscle (Figure 6E).

Subsequently, we performed a lipidomic analysis of muscle samples. FA18:1, FA16:0, FA18:2, FA16:1, FA18:0, w6‐FA18:3, w3‐FA18:3, FA20:1, FA20:2, and FA14:0 were the fatty acid fractions with the highest content, with the content of FA18:1 significantly increased by the injection, and the content of FA18:2 significantly decreased (Figure 6G and Table S4, Supporting Information). For the different fatty acid types, there was a significant increase in monounsaturated fatty acid (MUFA) and a decrease in polyunsaturated fatty acid (PUFA) content, while no significant change was detected in saturated fatty acids (SFA) (Figure 6H). Although it had been shown in mice that injection of OA‐aLDs did not lead to inflammatory responses and other tissue pathologies, we further tested porcine TNF‐α and IL‐1β using ELISA to exclude potential pathogenic effects. The results showed a significant decrease in TNF‐α and no significant change in IL‐1β. These results indicated that OA‐aLDs did not cause an inflammatory response (Figure 6I). As expected, the expression of Plin1, Plin2, Dgat2, Fasn, Pparγ, Srebf, and C/ebpα were upregulated at mRNA and protein levels, as validated by qPCR and western blot (Figure 6F,G). Notably, the expression of Seipin was also increased, which played a crucial role in the biogenesis of LDs. This suggests that lipid synthesis was stimulated in longissimus dorsi after injecting OA‐aLDs.

## 3 Discussion

IMF content is the most important indicator for evaluating pork quality and significantly affects meat tenderness, juiciness, and flavors. Since IMF content is difficult to measure both in living animals and at early developmental stages, conventional breeding is much more difficult to carry out. Improving IMF content while maintaining leanness has become a new challenge for breeders. Novel strategies to overcome these inherent challenges are urgently needed.

Methods to enhance lipid deposition by increasing dietary fatty acid uptake have been applied in livestock husbandry.^[^ ^24^, ^25^ ^]^ Changing the ratio of polyunsaturated fatty acids in the feed led to IMF deposition in goats and altered the expression of genes related to lipid metabolism, such as PPAR𝛼 and SCD.^[^ ^26^ ^]^ This was mainly achieved by adding different proportions of canola oil,^[^ ^27^ ^]^ sunflower oil,^[^ ^28^ ^]^ and soybean oil^[^ ^29^ ^]^ to feeds. Remarkably, a 4.5% canola oil addition significantly increased porcine IMF content, with the longest dorsal muscle even reaching 17.45%.^[^ ^30^ ^]^ However, livestock production is a systematic process and short‐term feeding may not lead to substantial fat deposition. Prolonged high‐fat diets resulted in overall fat deposition, especially subcutaneous and visceral fat, which adversely affected the reproductive and growth traits of livestock.^[^ ^31^, ^32^ ^]^

Genetic approaches have been extensively explored, with numerous studies identifying genes and single nucleotide polymorphisms associated with IMF deposition.^[^ ^33^ ^]^ However, many of the identified genes are expressed in both muscle and adipose tissues,^[^ ^34^ ^]^ limiting their ability to selectively enhance IMF while preserving carcass leanness. Breeding strategies based on these genes often result in excessive fat accumulation, limiting their practical applicability. Furthermore, the lack of targeted methods to increase IMF specifically hinders detailed comparative studies on the underlying mechanisms of IMF deposition.

IMF deposition essentially involves the accumulation of LDs in cells. We hypothesized that enhanced IMF could be achieved by directly introducing LDs into muscle cells. Synthetic biology offers a potential solution to our hypothesis. In this study, we demonstrated that LDs, after being loaded with OA, can serve as a highly biocompatible carrier to enhance IMF content when injected into muscle tissues. Injections of OA‐aLDs provided an increased effect in only 5 days compared to a minimum of one month of feeding on a high‐fat diet. The significant increase in IMF content observed in our study was due to the targeted delivery and controlled release capabilities of the nanocarriers. Targeting muscle tissue with this approach minimizes unintended systemic effects, a major limitation in traditional fattening methods. This is consistent with current research on nanolipid particles in targeted drug delivery,^[^ ^35^ ^]^ such as in cancer or gene therapy.^[^ ^36^, ^37^ ^]^ This rapid effect highlights the potential of nanocarriers as a transformative tool in livestock production.

The mechanism underlying this enhancement was based on the biological role of LDs in cellular lipid storage and metabolism. By synthesizing OA‐aLDs, we facilitated the direct uptake of OA into muscle cells, thereby activating PPARγ, a master regulator of adipogenesis. This activation promoted the transcription of genes involved in fatty acid and triglyceride synthesis, driving localized lipid accumulation. In addition, confocal imaging revealed extensive interactions between LDs and intracellular organelles, such as lysosomes and mitochondria, suggesting that these interactions play a crucial role in lipid metabolism and storage. Such interactions not only validated the bioactivity of OA‐aLDs but also provided a deeper understanding of the cellular processes underlying IMF deposition. Our approach also addresses a critical limitation in IMF research: the reliance on comparative studies between breeds or developmental stages, which are inherently influenced by genetic and environmental differences.^[^ ^26^, ^38^, ^39^ ^]^ By using local injections of OA‐aLDs for intra‐individual comparisons, our approach eliminates these variations and provides a more accurate model for studying the mechanism of IMF deposition. This capability fills a significant gap in this field and paves the way for more accurate and reliable studies.

However, our study still has some limitations that need to be further investigated. The long‐term safety of OA‐aLDs must be thoroughly evaluated by more extensive biochemical assays and histologic analyses. Additionally, the absorption efficiency and release kinetics of OA‐aLDs in vivo need to be optimized to improve their range and persistence. Currently, the action of OA‐aLDs is limited to the injection site, and improvements in the targeting mechanism could reduce injection frequency, making the method more applicable to large‐scale farming.

It is encouraging to note that aLDs are also lipid‐soluble carriers, and in addition to fatty acids, some lipophilic drugs can also be dissolved in aLDs. If OA‐aLDs can be combined with vaccination programs in the future, they could reduce operational procedures in the breeding process, providing dual benefits. This is also a potential approach, but the interaction between OA‐aLDs and the immune system on their efficacy needs to be confirmed first. In addition, the principles demonstrated in this study can be applied to the production of cell‐cultured meats,^[^ ^40^ ^]^ where OA‐aLDs can be used to enhance flavor and texture by delivering vegetable oils or other lipid additives. This combination of nanotechnology and animal science demonstrates the potential of cross‐field approaches to transform traditional agriculture.

## 4 Conclusion

In conclusion, our study demonstrated that OA‐aLDs can be used as a biomimetic delivery vehicle to increase intramuscular fat content in pigs. This study provides new insights into the production of high‐quality meat and a new theoretical basis for the development of drug carriers based on LDs.

## 5 Experimental Section

### Animals

The mice were purchased from the Animal Experiment Center of China Three Gorges University. Healthy 8‐week‐old male C57BL/6 mice were selected for feeding. For the gastrocnemius injection experiment, the mice were divided into 5 groups of 3 mice each, and the gastrocnemius muscle tissues were collected on days 0, 1, 3, 5, and 7 after the start of feeding. The volume of OA‐aLDs per injection was 10 µL. For each gastrocnemius muscle tissue, 6 frozen sections were prepared by making a transverse cut (perpendicular to the muscle fibers) at the central part of the tissue, and RNA or protein was extracted from the rest.

Twelve 100‐day‐old healthy male Tongcheng pigs (6 for the injection group, 3 for the feed group, and 3 for negative control) were selected for feeding and injected on days 1 and 3. The longissimus dorsi muscle was collected on day 5. The volume of OA‐aLDs per injection was 500 µL (2 times, 10 mg total lipid). For injection experiments, images were collected by transecting the entire muscle at 1 cm below the injection site. 6 frozen sections (1 cm × 1 cm × 8 µm) were prepared for each tissue, Pigs were purchased from the Experimental Pig Farm of Huazhong Agricultural University.

In order to be consistent with the lipid additions in the Injection group, 500 µL of OA‐aLDs (2 times, 10 mg total lipid) were added to the feed group diets at the same time points (Day 1 and 3). For carcass traits and meat quality evaluation, all pigs were fasted for 12 h and then transported to the Breeding Swine Quality Inspection and Testing Center, Ministry of Agriculture, and rural areas (Wuhan). The pigs were electrically stunned, exsanguinated, and eviscerated according to the standard commercial procedure. Carcass characteristics and meat quality evaluation were determined according to standard document requirements (NY/T 825–2004, NY/T 1180–2006, and NY/T 821–2004). The detailed measurement procedure followed the methods reported previously.^[^ ^36^ ^]^

### Materials

DOPC (1,2‐Di(9z‐octadecenoyl)‐sn‐glycero‐3‐phosphocholine) (D4250), Oleic acid (O0180), and triacylglycerol (T1392) were purchased from TCI, detailed structure is shown in Figure S7 (Supporting Information). BODIPY493/503 (D3922), BODIPY558/568‐labeled fatty acid C_12_ (D3835), ER‐Tracker (E34250), MitoTracker Red CMXRos (M7512) and LysoTracker (L7528) were obtained from Invitrogen. A922500 (HY‐10038), PF‐06424439 (HY‐108341A), chlorpromazine (HY‐12708), amiloride (HY‐B0285), and methyl‐β‐cyclodextrin (HY‐101461) were purchased from MedChemExpress.

### Preparation of aLDs

aLDs was prepared by methods previously reported.^[^ ^23^ ^]^ Briefly, triglycerides (300 mg) and DOPC (30 mg) were completely dissolved in ethanol, mixed with 30 mL of phosphate‐buffered saline (PBS), and stirred until the ethanol was completely evaporated to obtain the aLDs. The aLDs undergo morphological, biochemical, and functional analyses according to previous reports.^[^ ^37^ ^]^

For the OA gradient experiments, to ensure a consistent concentration of aLDs, 20‐fold concentrated OA‐aLDs according to the gradient was prepared. The preparation conditions were kept consistent except for the OA concentration, and the details of the preparation are shown in Table S2 (Supporting Information). 25 µL of OA‐aLDs were incubated in each 24‐well plate and 475 µL of the medium was added to perform subsequent experiments.

### Cell Culture

The C2C12 and C3H10 cell line was purchased from the Type Culture Collection of the Chinese Academy of Sciences (Wuhan, China). C2C12 and C3H10 cells were cultured in Dulbecco's modified Eagle's medium (DMEM; HyClone, Logan, UT, USA) with 10% fetal bovine serum (FBS; #SH30396.03, HyClone, Canada), 100 unit Ml^−1^ penicillin, and 100 µg mL^−1^ streptomycin in dishes at 37 °C in a humidified atmosphere with 5% CO2. Adipocyte differentiation media containing 0.5 µmol L^−1^ dexamethasone sodium (D‐085, Sigma, China), 50 mmol L^−1^ IBMX (410957, Sigma, China), and 0.5 µmol L^−1^ indomethacin (405268, Sigma, China) was replaced every 3 days.

### ROS and CCK8 Measure

According to the manufacturer's instructions, ROS generation was measured using a ROS assay kit (#S0033S, Beyotime Biotechnology, Nanjing, China). The fluorescence intensity was recorded on a PerkinElmer Enspire at Ex/Em = 488/525 nm. A CCK8 Kit (#A311‐01, Nanjing Vazyme Biotech) was used for the cell activity determination. Briefly, cells were prepared at a density of 2 × 103/well in 96‐well plates. After adding 10 µL of reagent for 2 h, the optical density at 450 nm was measured on a microplate reader (PerkinElmer, Germany).

### Antibodies and Immunoprecipitation

Rabbit polyclonal antibodies that were used included anti‐Plin2 (# A0270, Abclonal, Wuhan, China), anti‐DGAT2 (# A13891, Abclonal, Wuhan, China), anti‐FABP4 (# A11481, Abclonal, Wuhan, China), anti‐PPARγ (# A0270, Abclonal, Wuhan, China), and anti‐GAPDH (# A19056, Abclonal, Wuhan, China). The following secondary antibodies were used: HRP (horseradish peroxidase)‐labeled Goat Anti‐Rabbit IgG (H+L; #AS014, Abclonal, Wuhan, China). Western blot was performed as reported previously.^[^ ^38^ ^]^

### Isolation of Lipid Droplets

LDs were purified from C3H10 cells after OA treatment by methods previously reported.^[^ ^37^ ^]^ Briefly, cells were collected by adding 500 µL Buffer A (25 mm tricine, 25 mm sucrose, pH 7.8) containing 1 mm PMSF and incubated on ice for 30 min, and the cells were broken up using a homogenizer. The cells were then centrifuged at 16000 g for 1 h at 4 °C, and the upper white LDs were collected and washed with 500 µL of Buffer B (20 mm HEPES, 100 mm KCl, 2 mm MgCl2, pH 7.4) twice.

### Real‐Time qRT‐PCR

Total RNA was extracted using Trizol (# RK30129, Abclonal, Wuhan, China). First‐strand cDNA was synthesized using the ABScript II cDNA First‐Strand Synthesis Kit (# RK20400, Abclonal, Wuhan, China). Real‐time quantitative PCR was performed by Universal SYBR Green Fast qPCR Mix (# RK21203, Abclonal, Wuhan, China). Primers were used for amplification of target gene fragments respectively, PLIN1‐F (CAAGCACCTCTGACAAGGTTC); PLIN1‐R (GTTGGCGGCATATTCTGCTG); PLIN2‐F (CTTGTGTCCTCCGCTTATGTC); PLIN2‐R (GCAGAGGTCACGGTCTTCAC); PLIN3‐F (ATGTCTAGCAATGGTACAGATGC);PLIN3‐R (CGTGGAACTGATAAGAGGCAGG); PLIN4‐F (CGGCCCTTGTCGGAACTAAG); PLIN4‐R (CTTTGAAGTGTCAAGACCTCCC); ACSL3‐F (TGTCTTTCTCATGGATGCCGA); ACSL3‐R (CAGCACGGATGTGTCTCCTT); SREBF1‐F (GATGTGCGAACTGGACACAG); SREBF1‐R (CATAGGGGGCGTCAAACAG); FASN‐F (GGAGGTGGTGATAGCCGGTAT); FASN‐R (TGGGTAATCCATAGAGCCCAG); SCD1‐F (TTCTTGCGATACACTCTGGTGC); SCD1‐R (CGGGATTGAATGTTCTTGTCGT); PPARG‐F (GGAAGACCACTCGCATTCCTT); PPARG‐R (GTAATCAGCAACCATTGGGTCA); FSP27‐F (ATGGACTACGCCATGAAGTCT); FSP27‐R (CGGTGCTAACACGACAGGG); FITM1‐F (CCTCTGCCTTACTGTACTTTGG); FITM1‐R (TAGCGAAGATCGTCCGAGAGT); FITM2‐F (TCGGTCGTCAAGGAGCTGT); FITM2‐R (CAAAATACACGTTGAGGACGTTG); DGAT1‐F (GCCTTACTGGTTGAGTCTATCAC); DGAT1‐R (GCACCACAGGTTGACATCC); DGAT2‐F (CGAGACACCATAGACTACTTGCT); DGAT2‐R (GCGGTTCTTCAGGGTGACTG); FABP4‐F (AAGGTGAAGAGCATCATAACCCT); FABP4‐R (TCACGCCTTTCATAACACATTCC); HSL‐F (GATTTACGCACGATGACACAGT); HSL‐R (ACCTGCAAAGACATTAGACAGC); ATGL‐F (TCCGTGGCTGTCTACTAAAGA); ATGL‐R (TGGGATATGATGACGTTCTCTCC); MGL‐F (CGGACTTCCAAGTTTTTGTCAGA); MGL‐R (GCAGCCACTAGGATGGAGATG); GAPDH‐F (AGGTCGGTGTGAACGGATTTG); GAPDH‐R (TGTAGACCATGTAGTTGAGGTCA).

### Fluorescence Microscopy and Live Imaging

LDs or cells were incubated with BODIPY493/503 (1:1000 dilution), BODIPY558/568 (1:1000 dilution), or DAPI (1:1000 dilution) for 30 min at room temperature. Other dyes were used according to the manufacturer's instructions. Slides were sealed with an anti‐fluorescent quenching solution (#P36961, ProLong Diamond Antifade Mountant, Invitrogen, Thermo Fisher, USA) for confocal microscopic observation (Zeiss LSM 800, Germany). Three mice were used for in vivo imaging by injecting Rhod‐labeled OA‐aLDs into the gastrocnemius muscle tissues. Mice were anesthetized (R500IE, Rayward Life Technologies Inc, China) and photographed (IVIS Lumina LT Series III, PerkinElmer) every 24h.

### ELISA

Mouse IL‐6 Uncoated ELISA (88‐7064), Mouse TNF alpha Uncoated ELISA (88‐7324), Mouse IL‐1 beta Uncoated ELISA (88‐7013), Mouse IL‐10 Uncoated ELISA (88‐7105), Mouse C‐Reactive Protein/CRP ELISA Kit (EK294/2‐48, Liankebio), Porcine TNF‐alpha ELISA Kit (ES24RB, Thermofisher) and Porcine IL‐1 beta ELISA Kit (ESIL1B, Thermofisher) were used for ELISA detection. Muscle tissue was accurately weighed and add 9 times of the volume of homogenizing medium (0.86% or 0.9% normal saline was recommended) according to the proportion of weight (mg): Volume (UL): 1:9. Under the condition of ice water bath, mechanical homogenization was prepared into 10% homogenizing solution, 2500–3000 rpm, centrifugation for 10 min, and took the supernatant for determination. The assay procedure was carried out according to the instructions of the kit. The OD value of each well was measured at 450 nm using an enzyme meter. A standard curve was made based on the concentration and OD value of the standards, and then the sample concentration was calculated according to the standard curve equation.

### Lipidomics Analysis

Briefly, as previously described,^[^ ^39^ ^]^ lipids were extracted from ≈30 mg of frozen tissue by adding 150 µL of deionized water and 350 µL of chloroform to induce phase separation. The lower organic phase containing the lipids was extracted into a clean test tube, the lipid extraction was repeated once, and then the lipid extracts were pooled into a test tube and dried in the OH mode of the SpeedVac. Derivatization was carried out using 3‐nitrophenylhydrazine.^[^ ^41^ ^]^ Lipidomics analysis was performed by LipidALL Technologies Co., Ltd. (Changzhou, China).

### Encapsulation and Release Analyses

GC analyses were performed using a gas chromatograph (Thermo Scientific Trace 1610), with an HP‐88 capillary column (Agilent J&W). Nitrogen was used as the carrier gas at a flow rate of 3.0 mL min^−1^. The flame ionization detector (FID) was set at 280 °C, with hydrogen and airflow rates of 35 and 350 mL min^−1^, respectively, and helium as the makeup gas at 30 mL min^−1^. This method ensured precise detection and quantification of analytes. Briefly, the samples were hydrolyzed, extracted lipids, and subjected to saponification and methyl esterification, followed by content determination. The encapsulation efficiency of aLDs was calculated as the percentage of oleic acid (OA) successfully loaded into the lipid matrix relative to the total amount used during synthesis. The release efficiency was calculated by examining the ratio of OA in the solvent to the total OA content at 24h. The cumulative release rate was calculated by calculating the ratio of OA in the release medium (0.5% Tween80 in PBS) to the total encapsulated OA at 0, 6, 12, 24, and 48h.

### Cryo‐SEM

The aLD samples were prepared in liquid nitrogen and under vacuum. The water encapsulating the sample was sublimated and sprayed on the conductive layer. The samples were then placed on the cold stage of a scanning electron microscope via a cryogenic transport system for observation.

### Triglyceride Content Detection

The TG content was detected by TG measured kit (#A110‐1‐1, Jiancheng, Nanjing, China) following the manufacturer's recommendations. The collected cells were mixed with a working solution for 10 min. The optical density at 510 nm was measured on a microplate reader (PerkinElmer, Rodgau, Germany).

### Statistical Analysis

All quantitative experiments were evaluated for statistical significance using the software GraphPad Prism v.5.0 (GraphPad Software, Inc. 7825 Fay Avenue, Suite 230 La Jolla, CA, USA), after verifying the normality of values and equivalence of variances. Data are displayed as the means ± S.D. Sample size per statistical analysis (*n* ≥ 3). Statistical tests were performed with two‐tailed Student's t‐tests. A *p*‐value < 0.05 was considered statistically significant.

### Declarations—Ethics Approval Approval and Consent to Participate

All mouse mice and porcine were housed in a normal environment provided with food and water. The methods were performed in accordance with the approved guidelines from Huazhong Agricultural University, and scientific, ethical, and legal principles of the Hubei Regulations for the Administration of Affairs Concerning Experimental Animals. All experimental protocols were approved by the Ethics Committee of Huazhong Agricultural University.

## Conflict of Interest

The authors declare no conflict of interest.

## Author Contributions

P.Z. designed and wrote the main manuscript text. H.H, J.H, Z.Y, Z.Z and L.C. contributed to the specific sections. J.W. and Z.R. supervised and directed the overall project. H.W., J.W. and Z.R. reviewed and edited the manuscript. All authors contributed to the data analysis, and compilation, and reviewed the manuscript.

## Supporting information



Supporting Information

## Data Availability

Data sharing is not applicable to this article as no new data were created or analyzed in this study.
