# Supplementary material for: Lipid Droplet‐Derived Biomimetic Nanocarriers for the Enhancement of Porcine Intermuscular Fat Content
Source: Adv Sci (Weinh). 2025 May 14;12(21):2406150. doi: 10.1002/advs.202406150 (PMC12140346; doi:10.1002/advs.202406150)
Supplement: Supplementary file 1 — Supporting Information [file ADVS-12-2406150-s001.docx]

Supporting Information

**Lipid droplet-derived Biomimetic Nanocarriers for the Enhancement of Porcine Intermuscular Fat Content**

*Pengxiang Zhao^1,2^, Hongbo Han^1^, Jingjie Hao^1^, Ziwei Yu^1^, Zichen Zhao^1^, Lupeng Chen^1^, Heng Wang^1^, Jian Wu^1,3^, Zhuqing Ren^1,3,4, *^*

^1^Key Laboratory of Agriculture Animal Genetics, Breeding and Reproduction of the Ministry of Education, College of Animal Science, Huazhong Agricultural University, Wuhan, Hubei, P. R. China.

^2^College of Animal Science and Technology, Shandong Agricultural University, Taian, 271017, P. R. China.

^3^Frontiers Science Center for Animal Breeding and Sustainable Production, Wuhan 430070, China.

^4^Hubei Hongshan Laboratory, Wuhan, 430070, China

Figure S1 Stability of isolated LDs over 7 days in PBS.

Figure S2 Cumulative release rate of in vitro release for OA-aLDs.

Figure S3 Fluorescence density of C2C12 cell after incubating with aLDs. p<0.05.

Figure S4 Live imaging of mice after injection of Rhod-labeled aLDs

Figure S5 HE staining and fluorescence imaging of different tissues of mice.

Figure S6 Elisa of multiple inflammatory factors of mice after injection of OA-aLDs.

Figure S7 Their detailed structure of DOPC, OA and TAG.

Table S1 Routine blood tests in mice after injection of OA-aLDs.

Table S2 Preparation programs for different concentrations of OA-aLDs.

Table S3 Effect of OA-aLDs injection on carcass characteristics and meat quality of pigs.

Table S4 Descriptive statistics fatty acid composition in pig.


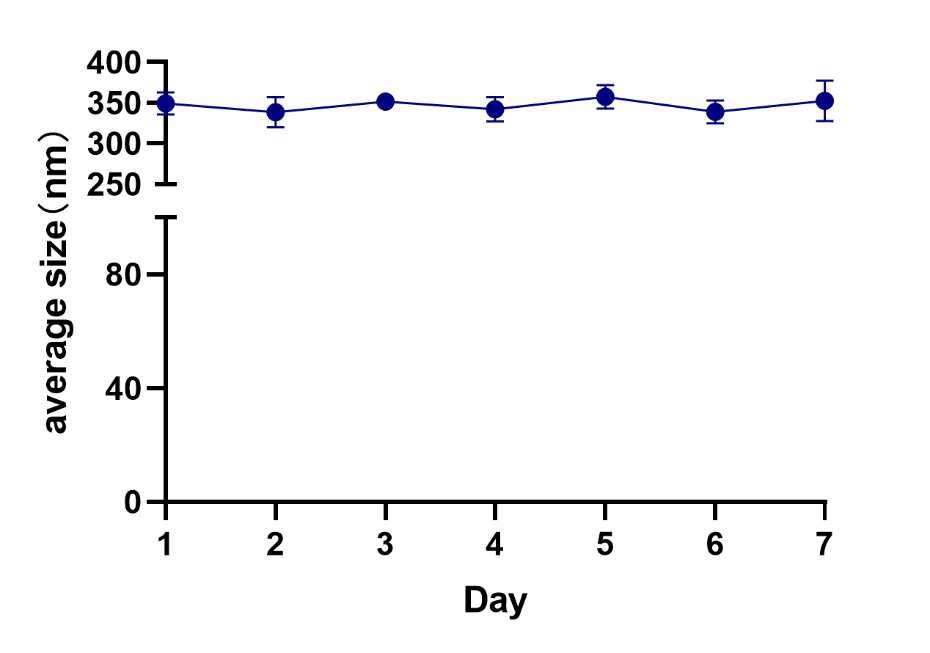


**Figure S1 Stability of isolated LDs over 7 days in PBS.**


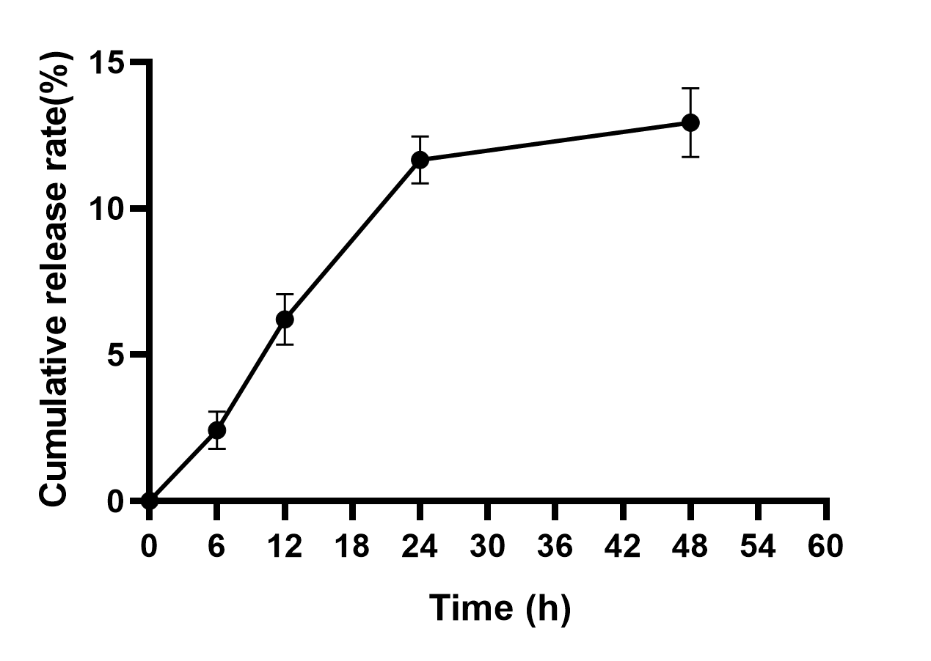


**Figure S2 Cumulative release rate of in vitro release for OA-aLDs.**


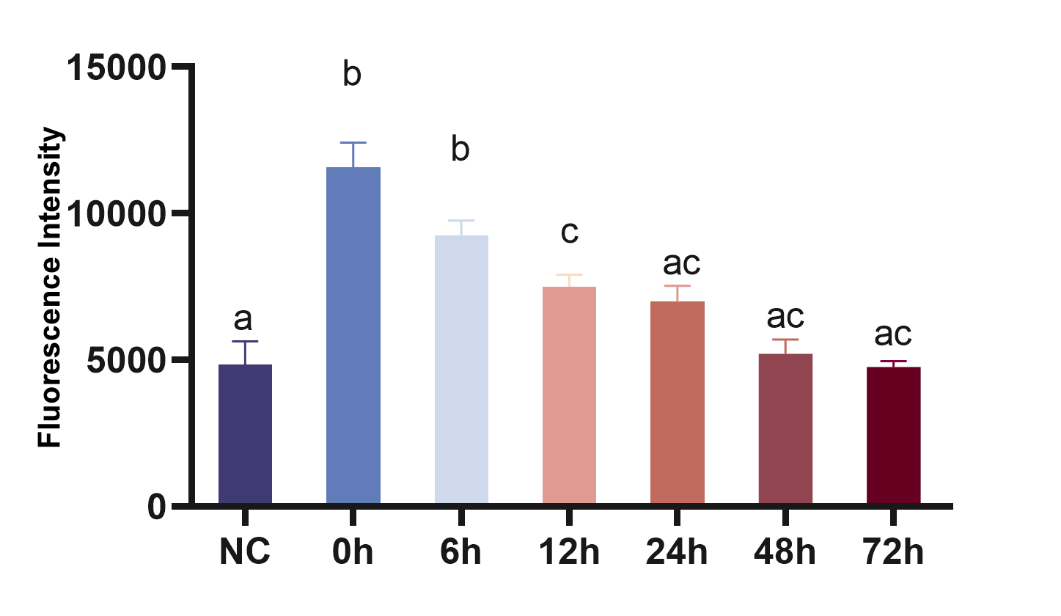


**Figure S3 Fluorescence density of C2C12 cell after incubating with aLDs. p<0.05.**


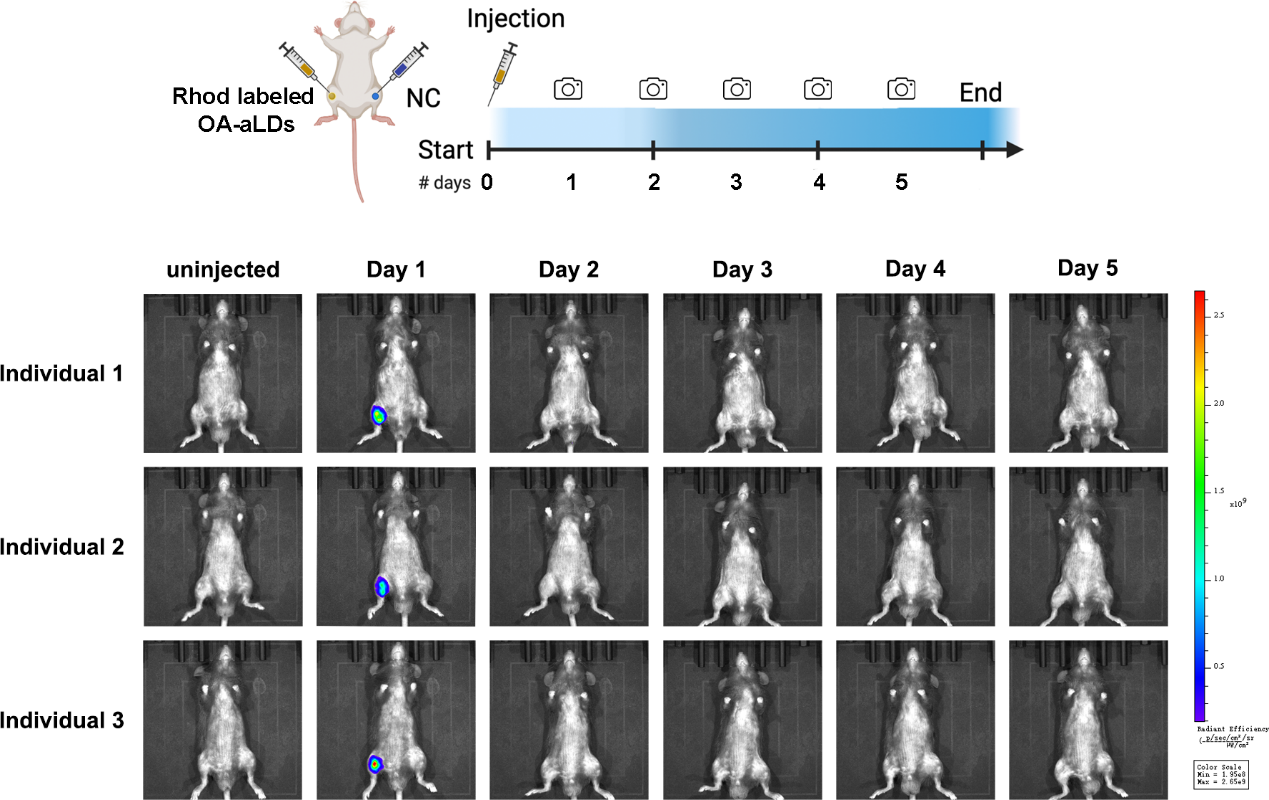


**Figure S4 Live imaging of mice after injection of Rhod-labeled aLDs**

Three mice were used for in vivo imaging by injecting Rhod-labeled OA-aLDs into the gastrocnemius muscle tissues. Mice were anesthetized and photographed every 24h.


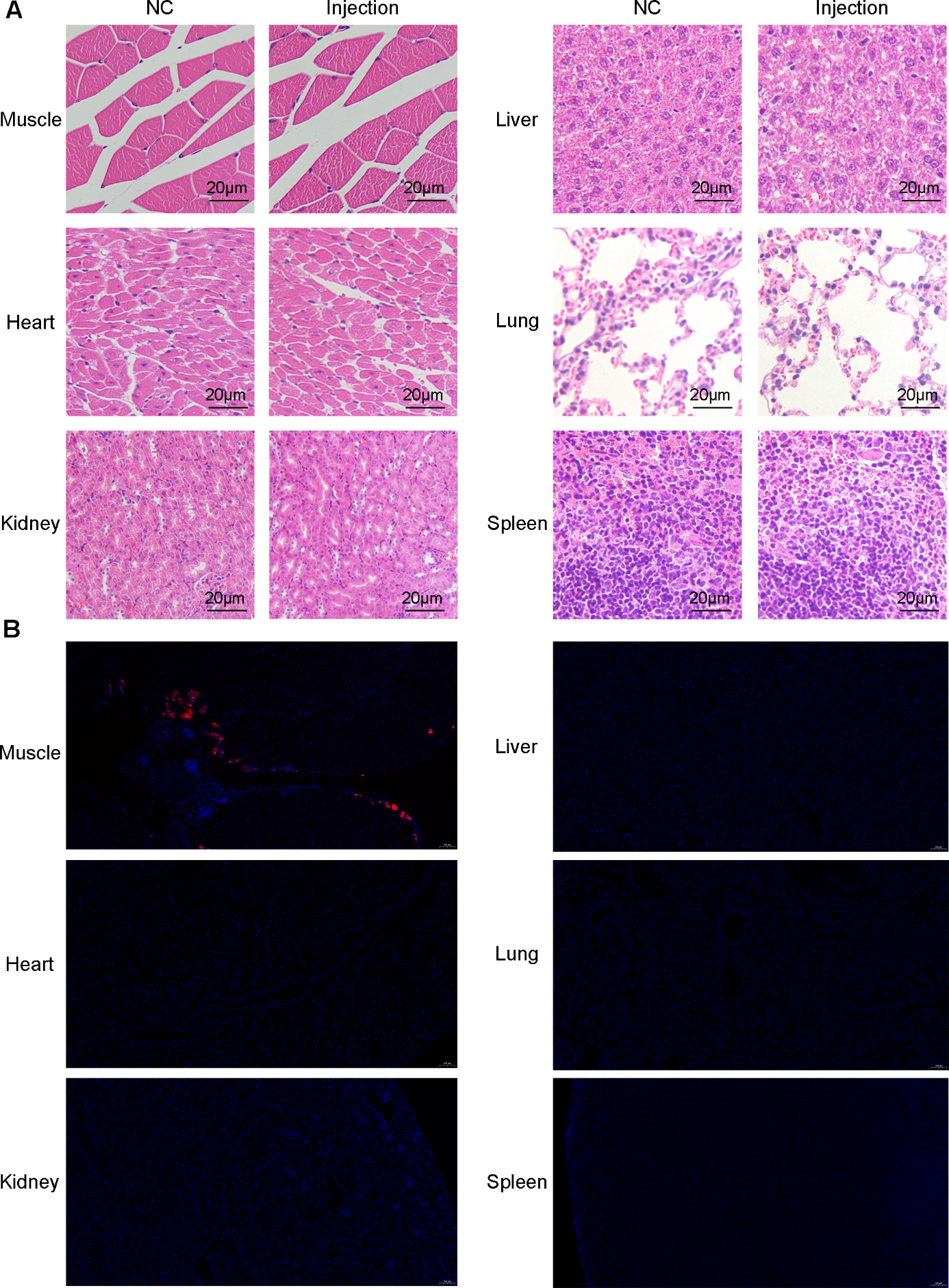


**Figure S5 HE staining and fluorescence imaging of different tissues of mice.** (A. HE staining of multiple tissues of mice after injection of OA-aLDs. B.Fluorescence imaging of multiple tissues of mice after injection of OA-aLDs.)


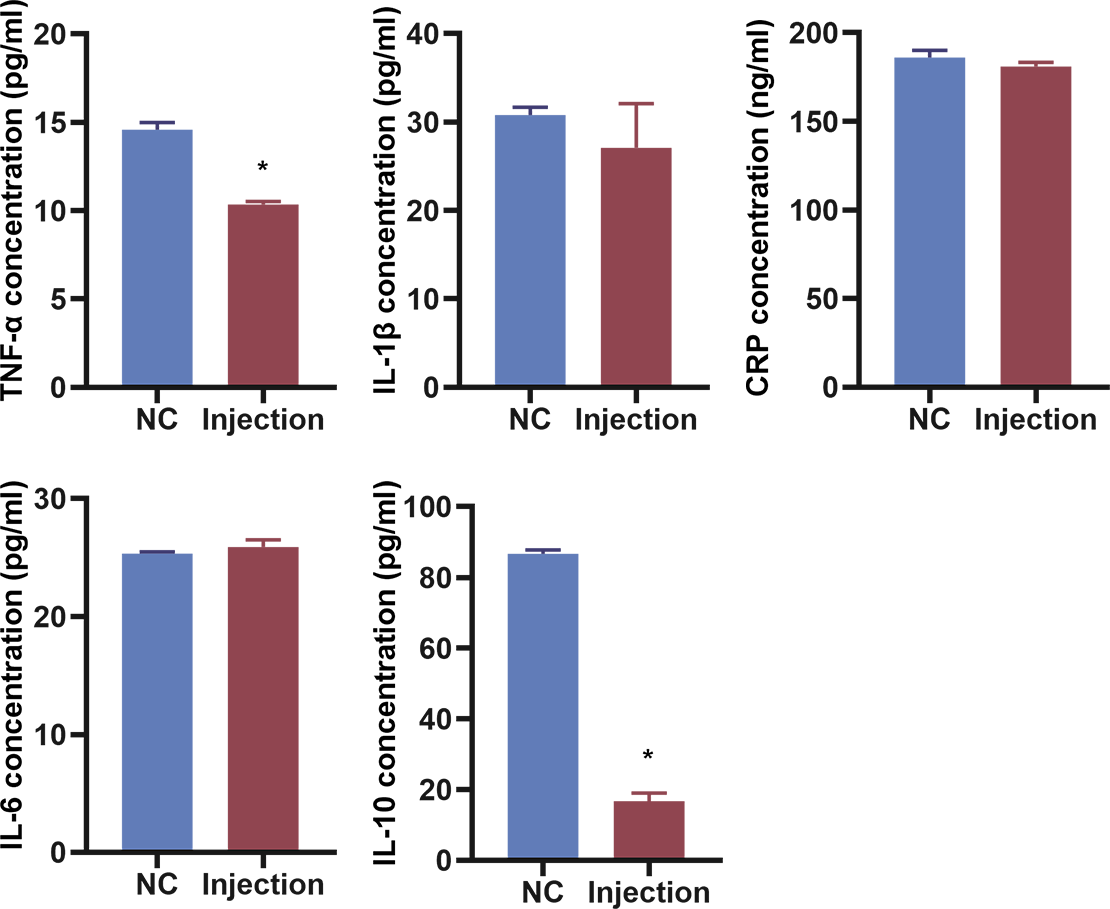


**Figure S6 Elisa of multiple inflammatory factors of mice after injection of OA-aLDs. *p<0.05.**

**Figure S7 Their detailed structure of DOPC, OA and TAG.**

Trielaidin

1,2,3-Tri(trans-9-octadecenoyl) Glycerol

CAS RN: 537-39-3


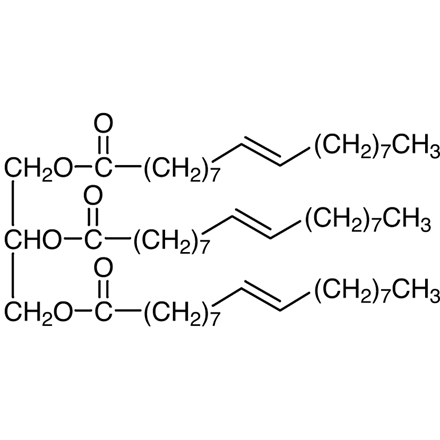


DOPC

CAS RN: 4235-95-4

1,2-Dioleoyl-sn-glycero-3-phosphocholine


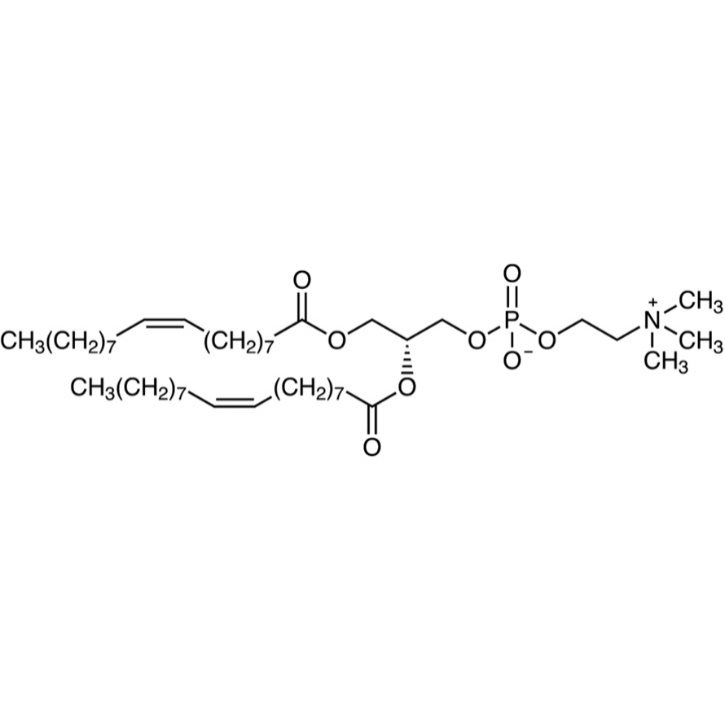


Oleic Acid

CAS RN: 112-80-1

cis-9-Octadecenoic Acid


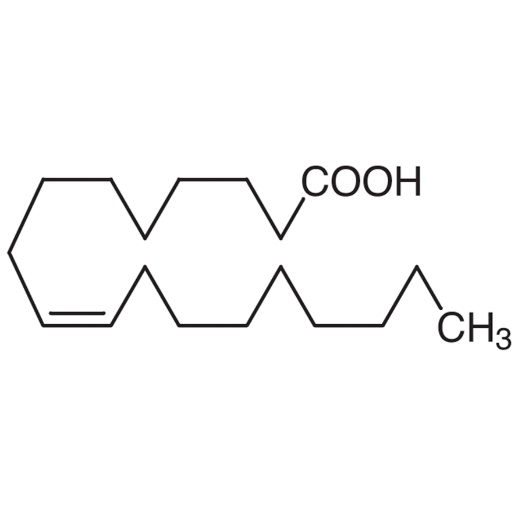


**Table S1 Routine blood tests in mice after injection of OA-aLDs.**

Routine blood tests of Mice after injection OA-aLDs (n=3)

|  | OA1 | OA2 | OA3 | Unit | Range |
| --- | --- | --- | --- | --- | --- |
| WBC | 6.2 | 3.5 | 5.8 | 10^9/L | 0.8-6.8 |
| Lymph# | 4.4 | 3 | 4.9 | 10^9/L | 0.7-5.7 |
| Mon# | 0.2 | 0.1 | 0.1 | 10^9/L | 0.0-0.3 |
| Gran# | 1.6 | 0.4 | 0.8 | 10^9/L | 0.1-1.8 |
| Lymph% | 70.9 | 84.6 | 84.1 | % | 55.8-90.6 |
| Mon% | 3.8 | 2.8 | 2 | % | 1.8-6.0 |
| Gran% | 25.3 | 12.6 | 13.9 | % | 8.6-38.9 |
| RBC | 9.36 | 7.81 | 9.14 | 10^12/L | 6.36-9.42 |
| HGB | 140 | 149 | 138 | g/L | 110-143 |
| HCT | 38 | 35.2 | 44.9 | % | 34.6-44.6 |
| MCV | 51.3 | 55.1 | 49.3 | fL | 48.2-58.3 |
| MCH | 16 | 19 | 15.9 | pg | 15.8-19 |
| MCHC | 312 | 423 | 324 | g/L | 302-353 |
| RDW | 14.5 | 16.5 | 14.7 | % | 13-17 |
| PLT | 1279 | 780 | 1572 | 10^9/L | 450-1590 |
| MPV | 4.4 | 5 | 5.7 | fL | 3.8-6.0 |

Routine blood tests for 3 normal mice

|  | NC1 | NC2 | NC3 | Unit | Range |
| --- | --- | --- | --- | --- | --- |
| WBC | 5.6 | 6.8 | 6.3 | 10^9/L | 0.8-6.8 |
| Lymph# | 4.1 | 5 | 4.8 | 10^9/L | 0.7-5.7 |
| Mon# | 0.2 | 0.2 | 0.2 | 10^9/L | 0.0-0.3 |
| Gran# | 1.3 | 1.6 | 1.2 | 10^9/L | 0.1-1.8 |
| Lymph% | 73.3 | 81.2 | 64 | % | 55.8-90.6 |
| Mon% | 2.9 | 2.6 | 5.4 | % | 1.8-6.0 |
| Gran% | 23.8 | 16.2 | 26.4 | % | 8.6-38.9 |
| RBC | 7.59 | 8.63 | 6.44 | 10^12/L | 6.36-9.42 |
| HGB | 144 | 137 | 127 | g/L | 110-143 |
| HCT | 41.8 | 42.7 | 37.5 | % | 34.6-44.6 |
| MCV | 57.5 | 49.5 | 58.3 | fL | 48.2-58.3 |
| MCH | 18.3 | 15.8 | 19.7 | pg | 15.8-19 |
| MCHC | 316 | 320 | 338 | g/L | 302-353 |
| RDW | 14.6 | 16 | 15.4 | % | 13-17 |
| PLT | 1524 | 474 | 1151 | 10^9/L | 450-1590 |
| MPV | 6.4 | 5.5 | 6 | fL | 3.8-6.0 |

**Table S2 Details of OA-aLDs preparation**

Details of OA-aLDs preparation

| Final concentration (μM) | 0 | 20 | 40 | 60 | 80 | 100 | 200 |
| --- | --- | --- | --- | --- | --- | --- | --- |
| Mass of OA (mg) | 0 | 3.39 | 6.78 | 10.17 | 13.56 | 16.95 | 33.90 |
| TAG (mg) | 300.00 | 296.19 | 292.38 | 288.57 | 284.77 | 280.96 | 261.92 |
| Mass of Total lipid (mg) | 330 | | | | | | |
| DOPC (mg) | 30 | | | | | | |
| PBS (mL) | 30 | | | | | | |
| OA-aLDs (20×) concentration (μM) | 0 | 400 | 800 | 1200 | 1600 | 2000 | 4000 |

To ensure the same concentration of aLDs, we performed cell incubation experiments using the following strategy

| Medium Volume (μL) | 475 | | | | | | |
| --- | --- | --- | --- | --- | --- | --- | --- |
| OA-aLDs (20×) added volume (μL) | 25 | | | | | | |
| final concentration of OA-aLDs (μM) | 0 | 20 | 40 | 60 | 80 | 100 | 200 |

**Table S3 Descriptive statistics of carcass characteristics and meat quality of pigs injected with OA-aLDs**

| Traits | Dietary treatment | | |
| --- | --- | --- | --- |
|  | Normal | Injection | Feed |
| Daily weight gain (g) | 682.6±144.5 | 716.8±146.4 | 715.5±233.3 |
| Feed Conversion Ratio (%) | 3.06±0.15 | 3.07±0.12 | 3.11±0.11 |
| Carcass weight (kg) | 37.22±4.91 | 38.13±1.60 | 35.84±3.95 |
| Back fat thickness (cm) | 16.70±2.93 | 16.77±1.32 | 16.07±2.29 |
| Lean meat percentage (%) | 53.60±1.98 | 57.53±2.50 | 56.37±5.65 |
| Fat percentage (%) | 16.97±2.65 | 16.57±0.12 | 15.90±6.61 |
| Bone percentage (%) | 14.50±0.90 | 14.40±2.01 | 14.85±1.51 |
| Loin eye area (cm^2^) | 21.47±1.38 | 21.39±1.20 | 19.75±1.45 |
| IMF (%) | 1.67±0.20^a^ | 2.15±0.12^b^ | 1.70±0.16^a^ |
| pH(1h) | 6.37±0.08 | 6.16±0.11 | 6.16±0.05 |
| pH(24h) | 5.71±0.09 | 5.66±0.03 | 5.69±0.06 |
| Water holding capacity (%) | 94.26±0.67 | 93.21±0.68 | 93.30±0.22 |
| Drip loss (%) | 2.70±0.24 | 2.76±0.20 | 2.71±0.04 |
| Marbling score | 2.00±0.00 | 2.33±0.47 | 2.00±0.00 |
| Tenderness (N) | 59.23±3.87 | 58.70±7.80 | 69.94±5.18 |
| Meat color | 3.50±0.00 | 3.50±0.00 | 3.50±0.00 |

Dietary treatment: Control: normal diet, injection: injection of OA-aLDs, Feed: diets supplemented with the same oleic acid content as the injection group.

^a, b^ Significantly different compared with two group at P < 0.05.

**Table S4 Descriptive statistics fatty acid composition in pig.**

| Triats | Mean (nmol/g) ± SD | |
| --- | --- | --- |
|  | OA-aLDs injection | Negative control |
| FA10:0 | 1.02 ± 0.18 | 0.86 ± 0.06 |
| FA12:0 | 1.64 ± 0.24 | 1.74 ± 0.13 |
| FA14:0 | 6.94 ± 1.5 | 5.69 ± 1.76 |
| FA15:0 | 1.64 ± 0.21 | 1.47 ± 0.13 |
| FA16:0 | 206.78 ± 33.27 | 176.2 ± 13.3 |
| FA16:1 | 58.22 ± 12.23 | 47.36 ± 5.44 |
| FA16:2 | 2.46 ± 0.8 | 1.84 ± 0.09 |
| FA17:0 | 3.87 ± 0.87 | 3.09 ± 0.31 |
| FA17:1 | 5.99 ± 1.3 | 4.75 ± 0.53 |
| FA18:0 | 57.55 ± 8.43 | 59.62 ± 5.7 |
| FA18:1 | 342.88 ± 74.75 | 278.86 ± 20.93 |
| FA18:2 | 126.73 ± 4.82 | 126.56 ± 5.02 |
| FA19:0 | 0.63 ± 0.05 | 0.55 ± 0.1 |
| FA20:0 | 1.61 ± 0.36 | 1.5 ± 0.47 |
| FA20:1 | 12.48 ± 0.8 | 11.53 ± 1.54 |
| FA20:2 | 8.19 ± 0.61 | 7.57 ± 0.47 |
| FA20:4 | 1.47 ± 0.29 | 1.52 ± 0.31 |
| FA20:5 | 7.54 ± 0.99 | 8.03 ± 0.34 |
| FA22:0 | 0.22 ± 0.16 | 0.32 ± 0.01 |
| FA22:3 | 0.88 ± 0.16 | 0.76 ± 0.01 |
| FA22:4 | 2.14 ± 0.12 | 2.03 ± 0.12 |
| FA22:6 | 4.69 ± 0.83 | 5.07 ± 0.34 |
| FA24:0 | 0.4 ± 0.11 | 0.41 ± 0.11 |
| FA24:1 | 0.83 ± 0.37 | 0.53 ± 0.06 |
| FA26:0 | 0.17 ± 0.09 | 0.2 ± 0.05 |
| FA26:4 | 1.57 ± 0.41 | 1.65 ± 0.3 |
| w3-FA18:3 | 17.58 ± 1.41 | 15.53 ± 2.02 |
| w3-FA22:5 | 1.77 ± 0.29 | 1.91 ± 0.11 |
| w6-FA18:3 | 18.67 ± 0.45 | 18.24 ± 0.59 |
| w6-FA22:5 | 4.45 ± 0.19 | 4.43 ± 0.21 |
